# The NUDGE trial pragmatic trial to enhance cardiovascular medication adherence: study protocol for a randomized controlled trial

**DOI:** 10.1186/s13063-021-05453-9

**Published:** 2021-08-11

**Authors:** Russell E. Glasgow, Christopher E. Knoepke, David Magid, Gary K. Grunwald, Thomas J. Glorioso, Joy Waughtal, Joel C. Marrs, Sheana Bull, P. Michael Ho

**Affiliations:** 1 Department of Family Medicine, University of Colorado Denver - Anschutz Medical Campus, Denver, USA; 2 Dissemination and Implementation Science Program of ACCORDS (Adult and Child Consortium for Health Outcomes Research and Delivery Science), Aurora, USA; 3 Department of Medicine, Division of Cardiology, University of Colorado Denver - Anschutz Medical Campus, Denver, USA; 4 ACCORDS (Adult and Child Consortium for Health Outcomes Research and Delivery Science), Aurora, USA; 5 University of Colorado Denver - Anschutz, Denver, USA; 6 Department of Biostatistics and Informatics, Colorado School of Public Health, University of Colorado Anschutz Medical Campus, Denver, USA; 7 U.S. Department of Veterans Affairs, Washington, DC USA; 8 mHealth Impact Laboratory Colorado School of Public Health, Aurora, USA; 9 Skaggs School of Pharmacy and Pharmaceutical Sciences, University of Colorado Anschutz Medical Campus, Aurora, USA; 10 Department of Community and Behavioral Health, Aurora, USA; 11 Digital Education, Denver, USA; 12 Department of Medicine, University of Colorado School of Medicine, Aurora, USA; 13 VA Eastern Colorado Health Care System, Aurora, USA

**Keywords:** Pragmatic trial, Medication adherence, Cardiovascular, mHealth, RE-AIM, Implementation, Dissemination, PRECIS-2

## Abstract

**Background:**

Nearly half of patients do not take their cardiovascular medications as prescribed, resulting in increased morbidity, mortality, and healthcare costs. Mobile and digital technologies for health promotion and disease self-management offer an opportunity to adapt behavioral “nudges” using ubiquitous mobile phone technology to facilitate medication adherence. The Nudge pragmatic clinical trial uses population-level pharmacy data to deliver nudges via mobile phone text messaging and an artificial intelligent interactive chat bot with the goal of improving medication adherence and patient outcomes in three integrated healthcare delivery systems.

**Methods:**

The Theory of mHealth, the Expanded RE-AIM/PRISM, and the PRECIS-2 frameworks were used for program planning, implementation, and evaluation, along with a focus on dissemination and cost considerations. During the planning phase, the Nudge study team developed and piloted a technology-based nudge message and chat bot of optimized interactive content libraries for a range of diverse patients. Inclusion criteria are very broad and include patients in one of three diverse health systems who take medications to treat hypertension, atrial fibrillation, coronary artery disease, diabetes, or hyperlipidemia. A target of approximately 10,000 participants will be randomized to one of 4 study arms: usual care (no intervention), generic nudge (text reminder), optimized nudge, and optimized nudge plus interactive AI chat bot. The PRECIS-2 tool indicated that the study protocol is very pragmatic, although there is variability across PRECIS-2 dimensions.

**Discussion:**

The primary effectiveness outcome is medication adherence defined by the proportion of days covered (PDC) using pharmacy refill data. Implementation outcomes are assessed using the RE-AIM framework, with a particular focus on reach, consistency of implementation, adaptations, cost, and maintenance/sustainability. The project has limitations including limited power to detect some subgroup effects, medication complications (bleeding), and longer-term outcomes (myocardial infarction). Strengths of the study include the diverse healthcare systems, a feasible and generalizable intervention, transparent reporting using established pragmatic research and implementation science frameworks, strong stakeholder engagement, and planning for dissemination and sustainment.

**Trial registration:**

ClinicalTrials.govNCT03973931. Registered on 4 June 2019. The study was funded by the NIH; grant number is 4UH3HL144163-02 issued 4/5/19.

**Supplementary Information:**

The online version contains supplementary material available at 10.1186/s13063-021-05453-9.

## Contributions to the literature

- This protocol addresses the implementation and effectiveness of a unique text- and phone-based medication adherence program across different cardiovascular conditions and types of healthcare settings.
- This project develops technology-based mHealth “nudge” messages using longitudinal engagement of multiple stakeholders including patients, physicians, pharmacists, and administrators.
- Evaluation is conceptually based using the updated Expanded RE-AIM/PRISM framework.
- We illustrate program and design planning using PRECIS-2 and designing for dissemination and sustainment strategies to produce a pragmatic trial.
- The balanced inclusion of intervention fidelity assessment, study of adaptations, and detailed cost assessment addresses important issues for implementation science.

## Background

Up to 50% of patients do not take their cardiovascular (CV) medications as prescribed [1–6], resulting in increased morbidity, mortality, and healthcare costs [4, 7, 8]. Interventions to improve adherence, such as patient education, reminders, pharmacist support, and financial incentives, have produced mixed results—some demonstrating benefits, but many producing small to negative results [9–14]. Adherence interventions have been limited by (1) including adherent patients who may not need an intervention; (2) resource-intensive approaches involving pharmacists or behavioral health providers, often working outside their normal scope of work; and (3) lack of attention to evidence-based strategies to motivate behavior change [6].

Brief behavioral interventions can influence decision-making and are impactful. Principles of behavioral economics have been incorporated into health interventions to “nudge” people to achieve improved health outcomes [15]. A behavioral nudge is a small change in framing choice that alters people’s behavior in a predictable way. A prior study testing financial incentives through elimination of copayments for cardiovascular medications in the year after acute myocardial infarction improved adherence by 4 to 6%; however, financial incentives are not generalizable and are unlikely to be sustainable [10, 16]. Behavioral nudges such as commitments (e.g., asking patients for demonstrated commitment to change through a pledge), norms (using examples of others who take action), and salience (making information or recommendations resonant through use of stories) build on a well-evidenced body of behavioral science theory and have been shown to improve health behaviors such as smoking cessation and weight loss [15, 17]. These have yet to be tested to improve medication adherence.

Mobile and digital technologies for health promotion and disease self-management [18–20] offer intriguing and yet untested opportunities to adapt behavioral “nudges” using ubiquitous cell phone technology to facilitate medication adherence. As described below, the NUDGE study contains several elements to make it pragmatic, including applying the PRECIS-2 criteria and the Expanded CONSORT diagram [21, 22]. These include diverse health systems and a large and diverse number of patients to evaluate generalizability. There are very few exclusion criteria and the intervention is largely automated. Measures include those important to health systems and patients and most are available using unobtrusive measures such as electronic health record data and tracking of study implementation activities.

The objectives of the multi-center NUDGE study are as follows over the course of 4 years:

1. Conduct a pragmatic patient-level randomized intervention across three healthcare systems (HCS) to improve adherence to chronic CV medications. The primary effectiveness outcome will be medication adherence defined by the proportion of days covered (PDC) using pharmacy refill data. Secondary effectiveness outcomes will include intermediate clinical measures (e.g., BP control), CV clinical events (e.g., hospitalizations), and healthcare utilization.
2. Evaluate the intervention and implementation using a mixed methods approach and applying the Expanded RE-AIM (reach, effectiveness, adoption, implementation, and maintenance) framework. Key implementation outcomes will include reach, implementation consistency and adaptations, costs, and program maintenance. In addition, NUDGE assess the context and implementation processes to inform local tailoring and eventual expansion of the intervention within the three HCS more broadly and nationally.

## Methods

### Study settings

The study will be conducted within three HCS: the VA Eastern Colorado Health Care System, the Denver Health and Hospital Authority, and the University of Colorado UCHealth. Patient, provider, and health system stakeholders affiliated with each participating health system provided input on the development of the project and have committed to participate in all aspects of the study in the pragmatic clinical trial phase.

### VA Eastern Colorado Health Care System

The VA Eastern Colorado Health Care System (VA) is comprised of the Rocky Mountain Regional (RMR) VA Medical Center (Aurora, Colorado) and its affiliated clinics. The health system is comprised of 12 community-based outpatient care clinics throughout the state. Our study will enroll patients from eastern Colorado. The VA Health Care System has a single electronic health record (i.e., Computerized Patient Record System [CPRS]) and a centralized corporate data warehouse (CDW) that houses clinical and pharmacy data. It is estimated that ~ 70% of VA patients obtain their medications through VA pharmacies. The rest get them through outside pharmacies.

### Denver Health and Hospital Authority

Denver Health (DH) is an integrated healthcare system that serves as the primary healthcare safety net for the City and County of Denver and serves an estimated one in four Denver residents, or 208,000 people, per year. Our study will enroll patients from primary care clinics located throughout the Denver Metro region. The DH technology infrastructure includes an EPIC electronic health record (EHR) and a data warehouse that integrates patient clinical and administrative data, including pharmacy data. It is estimated that approximately two-thirds of DH patients obtain their medications through DH pharmacies.

### UCHealth

UCHealth (formally known as the University of Colorado Health) currently includes 11 hospitals across the state, hosting over three million annual outpatient visits and covering 19% of Coloradans plus referrals from surrounding states. Our study will enroll patients from primary care clinics located in the Denver Metro region. The system’s infrastructure includes a single instance of the EPIC EHR for all inpatient and outpatient activities. Patients currently get their medications at both UCHealth pharmacies and pharmacies outside of UCHealth. Our plan is to get medication refill data through Surescripts, which is a health information technology company that supports *e-prescription* and the electronic transmission of prescriptions between healthcare organizations and pharmacies. Surescripts provides information to UCHealth about medication filled outside of the health system.

**Fig. 1:**
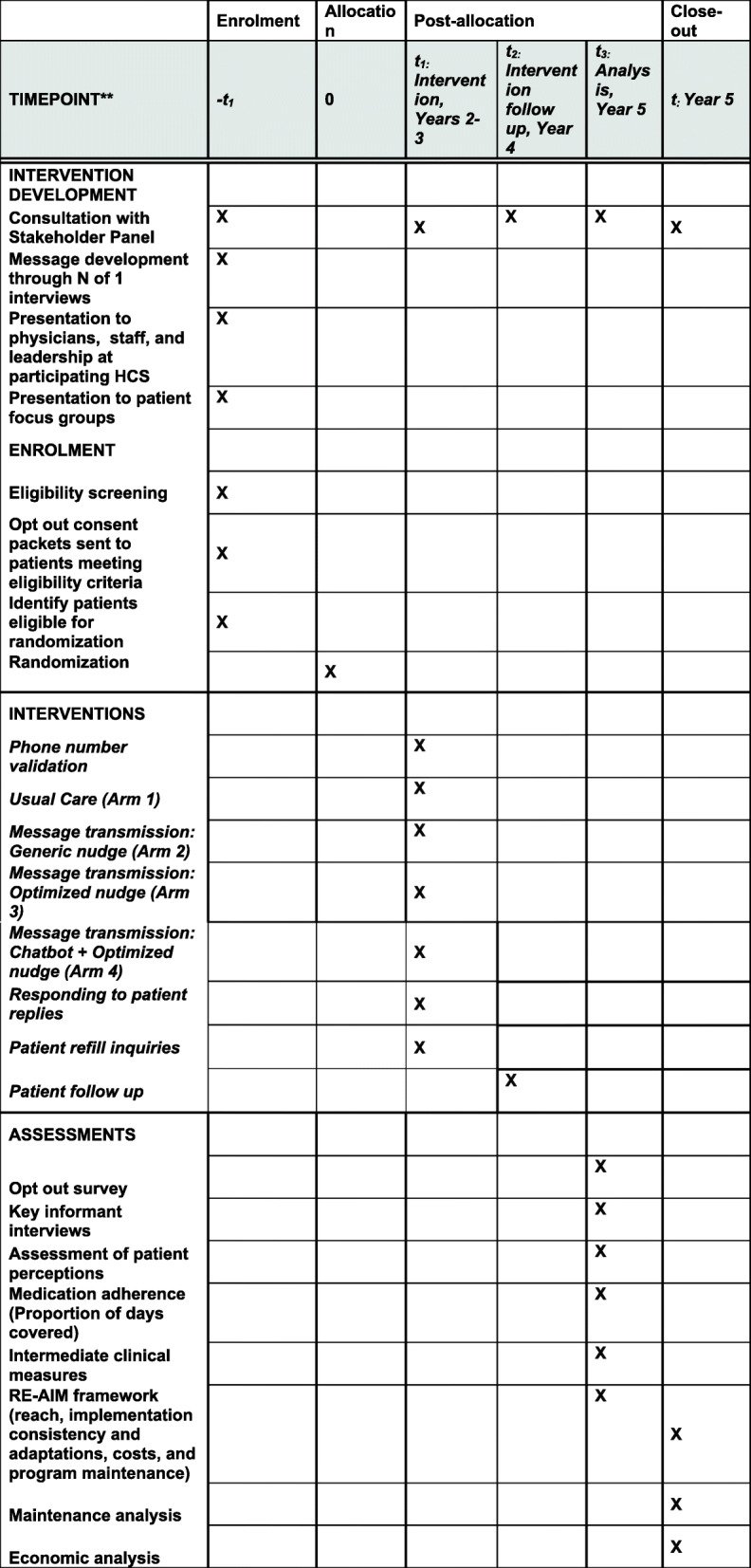
SPIRIT checklist outlining the schedule of enrolment, interventions, and assessments

All development, enrollment, allocation, and analytic features are outlined in Fig. 1.

### Patient population

#### Identifying patients with clinical conditions and prescribed medication classes of interest

Designated HCS programmers identify eligible patients based on the presence at least 1 of the cardiovascular conditions listed in Table 1 and with a filled prescription for at least one of the classes of medications to treat these cardiovascular conditions within 100 days prior to study eligibility. Participants must have had a primary care clinic visit at one of the clinics at UCHealth or Denver Health, and within 2 years (VA; due to differences in the electronic health record (EHR) systems) of the time of the data pull.

**Table 1:** Inclusion criteria, cardiovascular conditions, and medication classes

Condition	Classes of medications
Hypertension	Beta-blockers (B-blockers), calcium channel blocker (CCB), angiotensin-converting enzyme inhibitors (ACEi), angiotensin receptor blockers (ARB), thiazide diuretic
Hyperlipidemia	HMG CoA reductase inhibitor (statins)
Diabetes	Alpha-glucosidase inhibitors, Biguanides, DPP-4 inhibitors, sodium glucose transport inhibitor, Meglitinides, sulfonylureas, thiazolidinediones, and statins
Coronary artery disease	PGY-2 inhibitor (Clopidogrel, Ticagrelor, Prasugrel, Ticlopidine), B-blockers, ACEi or ARB and statins
Atrial fibrillation	Direct oral anticoagulants, B-blockers, CCB

International Classification of Diseases, Ninth and Tenth Revision (ICD 9 and 10) codes identifying the clinical conditions of interest were compiled by each participating HCS. National Drug Codes (NDC) or system-specific medication codes were used to identify the medication classes of interest. On a quarterly basis, we identify new patients who have met eligibility criteria based on the clinical condition and filling one of the classes of medication of interest.

Exclusion criteria were purposefully kept minimal to maximize generalizability including patients who (1) do not have a mailing address listed in EHR, (2) do not have a landline or cell phone listed in EHR, (3) are currently pregnant if denoted in the EHR at the time of the data pull, or (4) have a mailing address outside of the state of Colorado, (5) primarily communicate in a language other than English or Spanish, and (6) have a referral to hospice or palliative care.

**Fig. 2:**
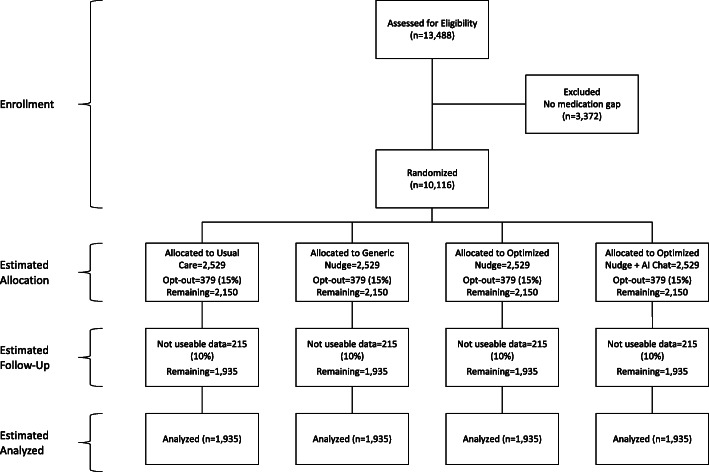
CONSORT figure and estimated participation and attrition rates in the Nudge trial

All eligible patients identified above are sent an opt-out consent packet. The packet contains an introductory letter with information about the study, an opt-out form, an opt-out survey, and a self-addressed, stamped envelope. We will send materials to patients in their preferred language if that preference is denoted in the EHR (i.e., English or Spanish). All materials will be sent on letterhead and branding appropriate and specific to each HCS. The letter will be signed by either the primary care provider for the patient or the site principal investigator. We will provide patients with 4 weeks to return the opt-out postcard from the date that the introductory study packet was sent out. If they did not return the opt-put postcard by this period of time, the patient is eligible for the study. The CONSORT diagram in Fig. 2 shows our anticipated sample size, participation, and attrition rates.

#### Identifying patients eligible for randomization

Patients meeting the above screening criteria are followed prospectively for pharmacy refill gaps using HCS-specific pharmacy data. Daily pharmacy data are obtained within each HCS with prescription information including a patient identifier, medication name, medication class, release date, and days’ supply for each fill. We determine a date that each medication class is due to be refilled (expected refill date) for each patient. This expected refill date is calculated as the date of the last fill for a specified medication class plus its days’ supply. We adjust this expected refill date after considering factors such as medication supply on-hand using information up to 6 months prior to study eligibility, inpatient days assuming the medication is provided to the patient in this setting, and cancelation of a medication.

Patients identified to have 7 or more consecutive days without filling a medication after this expected refill date (7-day gap) for a medication class listed in Table 1 at any time during the 2-year monitoring period will be randomized. For patients who are prescribed multiple CV medications, eligibility for randomization will be triggered by the first 7-day gap for any medication. Once the study starts at a clinic, we randomize all patients who currently have a medication refill gap of 7 or more days. Once randomized, patients will remain in the same study arm for the entire study whether or not they have subsequent refill gaps.

#### Randomizing patients who meet eligibility criteria

As part of the automated daily tracking of medication data, patients are identified as having an initial 7-day gap necessary for enrollment. Once identified, we determine the randomization stratum based on the HCS and number of baseline medications and then randomize patients to 1 of 4 study arms: (1) usual care, (2) generic text, (3) nudge text, and (4) nudge text with AI chat bot. Stratified randomization based on HCS as well as the number of baseline medication classes increases the likelihood of balance across groups for these key variables.

Patients randomized to the usual care arm will not receive further study procedures. For patients randomized to study arms 2–4, we determine if the listed phone number is a landline or cell phone using a validation process available as part of our text messaging platform (Mobile Messenger; Upland Communications, Austin, TX). Patients with a landline phone number will receive interactive voice responses (IVR; processes outlined below) automated messages instead of text messages. All procedures were reviewed and approved by the Colorado Institutional Review Board on April 9, 2019. Patient recruitment began Nov. 11, 2019, and is estimated to be completed approximately July 30, 2022.

### Pragmatic trial design

**Fig. 3:**
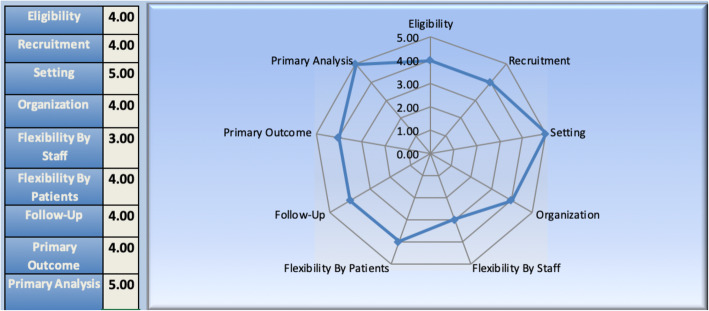
PRECIS-2 figure

To assess the level of pragmatism of the trial as designed, we engaged several investigators in a three-step sequential evaluation guided by the Pragmatic Explanatory Continuum Indicator Summary (PRECIS-2) [21, 23], using methods previously designed by our group. In the first stage of evaluation, the co-principle investigators, as well as two co-investigators and a staff member with expertise in pragmatic trial design, met to formally discuss the definitions of each PRECIS domain, taking care to specifically avoid discussion of how each would be scored in the Nudge trial. In the next phase, all four investigators independently scored the Nudge protocol along each dimension, returning their scorecards to the staff member without consulting with each other about scores. Finally, the investigators met once more to adjudicate final scores within each domain, discussing the merits and nuances of each until a final integer score was reached (Fig. 3). In general, Nudge is designed as a highly pragmatic trial, with consensus rankings across domains. The only domain scoring below a “4” was “Flexibility by Staff,” which was scored as a “3” owing to a moderately rigid conceptualization of how and when intervention is delivered to participating individuals.

### Interventions and implementation strategies

#### Intervention development

The Nudge interventions and implementation strategies were developed using our Integrated Theory of mHealth framework and “designing and disseminating” principles [24–26]. This involved rapid, iterative, user-centered design procedures, and the resulting messages and delivery processes were then piloted and adapted to result in the procedures below.

### Treatment conditions

1. Usual care: This group will not receive an intervention. We have included a usual care group to demonstrate the impact of the text messaging interventions above and beyond usual care and changing context, given that many prior medication adherence interventions have demonstrated small to negligible effects.
2. Generic nudge: A generic reminder text (see below) is delivered to patients to refill their medication at days 1, 3, 5, 7, and 10 after randomization.
3. Optimized nudge: A behavioral nudge text (see below) is delivered to patients to remind them to refill their medications at days 1, 3, 5, 7, and 10 after randomization.
4. Optimized nudge plus AI chat bot: A behavioral nudge text is delivered to patients to remind them to refill their medications at days 1 and 3 after randomization. If the patient has not filled their medication on days 5 and 9, in addition to receiving a behavioral nudge text, an AI algorithm delivers an interactive chat via a chat bot to assess barriers filling the medication.

**Fig. 4:**
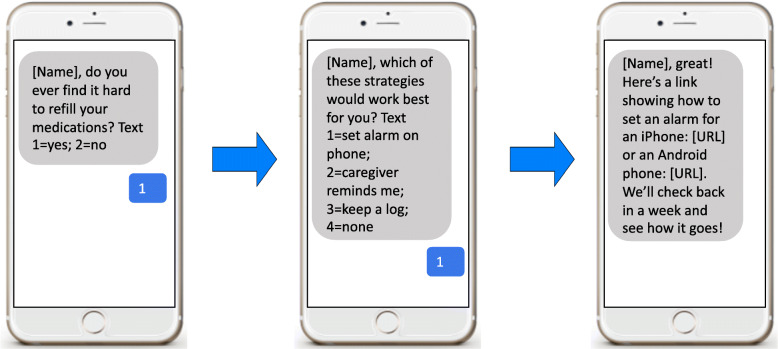
Example of text messages

The AI chat bot assesses for common barriers to medication adherence: (1) social determinant factors, (2) provider-patient/healthcare system factors, (3) condition-related factors, (4) therapy-related factors, and (5) patient-related factors using a script. Communication about all these barriers is pre-programmed to use as algorithms in the chat bot automated program. For each barrier, the AI chat bot problem-solves with the patient and identifies commonly used successful approaches to overcome barriers and asks patients to choose and enact one solution to improve medication adherence. The AI chat bot library includes algorithms to support specific strategies to circumvent the adherence barriers responsible for each instance of a medication refill gap. For example, patients are queried to determine if they have difficulty remembering what medications to take and when to take them; those that do are asked if using a medication diary, involving a caretaker, or setting an alarm on their phone would help. Patients are asked if they would like to try one of these strategies; for those that agree and identify a strategy, the AI chat program includes an algorithm to check in 1 week later to see how this strategy is going. This is illustrated in Fig. 4.

### Message transmission

Patients with a cell phone number will receive text messages according to their assigned study arm via Mobile Messenger, an online platform specializing in text message transmission. Patients with a landline phone number (estimated at 5–10%) receive messages via (IVR) automated telephone messages. The IVR calls follow the same flow logic, message content, and frequency of calls as the text messages. They remain in the same study arm for the duration of the study and receive the same intervention for subsequent episodes of 7-day medication gaps.

### Responding to text messages from patients

If a patient texts “stop” to unsubscribe, they are automatically and fully withdrawn from the study. Should a patient respond “done” to indicate they have already filled their prescription, we will hold sending patients any further text messages about refilling the medication in which they had a gap for 30 to 90 days based on the medication. If the patient has a refill gap for the same or another medication again, we will start delivering text messages within the same study arm to which they had been previously randomized. Patients may request Spanish messages at any time via text. We will start to deliver Spanish language texts following the request.

There will be text messages that do not fall into any of the categories above. A research assistant (RA) will monitor these responses and will triage the messages depending on the content of the messages. In our pilot study, some patients sent responses to the text messages that (a) requested additional information about the study and/or (b) requested more detail on the specific medication that required a refill, even though the text message they were responding to did not solicit this information. For these types of unsolicited messages, the RA will respond with a link to our study webpage where we will post information about the study, sponsors, participating institutions, and a contact number they can call for more information. This webpage will include a “Frequently Asked Questions” (FAQ) about Nudge link, and we will post responses to anticipated questions there (e.g., “Does my provider know about this study?; How did you get my cell number?; What if I don’t want to participate?”; etc.)

Patients may text unsolicited information about a side effect or adverse event related to their medications. In these cases, we will have the site study pharmacists call the patient to find out more about the issue. We will also have the pharmacist contact the patient’s provider to make them aware of the issue. Other responses, such as questions about the intervention, requesting information about their medication, or asking about the cost or logistics of obtaining the medication, will by triaged and responded to by a RA, pharmacist, or physician, as deemed appropriate. We will catalogue the messages that we receive from patients, and if there is a theme, we will develop a FAQ and place information on the study website.

### If patients do not refill after the series of text messages

In the cases where the patient still has not refilled their medication after 5 days of their reply, a study RA will first confirm that the medication refill has not been completed. If the medication has not been refilled after chart review, they will then contact the patient to see if there are having issues with refilling the medication and try to resolve any issues with the patient. The RA will follow the following script when contacting the patient. As this is a pragmatic trial conducted in the context of usual care, concomitant patient care will be tracked, but will be permitted.

## Study evaluation

The study will be evaluated using the Practical, Robust Implementation and Sustainability Model (PRISM) and its component RE-AIM framework, and mixed methods assessments to identify key contextual factors and RE-AIM framework evaluation components of Reach, Effectiveness, Adoption, Implementation, and Maintenance [27]. We will also develop tools and a sustainability plan to broadly disseminate and guide the intervention, if the intervention is found to be efficient. PRISM considers important implementation concepts from Diffusion of Innovations [28], the Chronic Care Model [29], the Model for Improvement [30], and the RE-AIM framework [31] and highlights four components that influence implementation success: (1) organizational and participant characteristics, (2) intervention characteristics from the organizational (healthcare system and providers) and participants’ perspectives (i.e., patients), (3) implementation and sustainability infrastructure (e.g., training and support, job roles, audit and feedback systems), and (4) external environment (e.g., reimbursement policies, guidelines). These four elements will be assessed in a formative manner [32] and will be critical to understanding how to further disseminate the intervention if demonstrated to be efficient. We will incorporate the assessment of the four components that influence RE-AIM implementation outcomes into our evaluation and this is further discussed in the implementation evaluation below (see Table 2).

**Table 2:** RE-AIM outcomes

A. RE-AIM dimension	B. Dimension description	C. Measure	D. Data source
**Reach**	Degree to which target population is impacted	1. Number of eligible patients (% patients with a 7-day gap) 2. % of patients who did not opt out 3. Representativeness of study participants compared to overall patients within each respective health system 4. Reasons why patients decline	Study database derived from EHR clinical and pharmacy data EHR data; also, brief phone interview with those who decline (if permission given on opt-out form?)
**Effectiveness**	Success of the intervention in changing patient outcomes	1. Improvement in medication adherence (PDC) and reduction in utilization/clinical outcomes/costs 2. Generalization (heterogeneity) of effects across patient subgroups 3. Unintended consequences—either positive or negative	Study database. Analytic plan for the primary outcome of interest is further discussed in the analytic plan
**Adoption**	Degree to which interventions are taken up by organizations, clinics, providers, and pharmacists	1. Records of clinics, physicians, and pharmacists approached and willingness to participate in the intervention 2. Clinic, physician, and pharmacist characteristics of those participating vs. not—if < 90% participate 3. Reasons for declining	Study database Brief phone interviews with subset of those who decline
**Implementation**	Degree to which interventions are implemented as intended (fidelity). (a) Adaptations made; (b) costs; and (c) contextual factors associated with outcomes	1. Among patients with gap, how many interventions were delivered per patient 2. Proportion and representativeness of those reached and by method (text message versus IVR) 3. Among patients in arm #4, proportion where AI chat bot was used, and the barrier identified 4. Mixed methods assessment of adaptions (see above) 5. Budget impact/cost of the program and replication costs (see below) 6. Qualitative interviews focused on PRISM factors of (1) organizational and participants characteristics, (2) intervention characteristics from the organizational (healthcare system and providers) and participants’ perspectives (i.e., patients), (3) implementation and sustainability infrastructure (training and support), and (4) external environment	1. Qualitative interviews 2. Study database 3. Health economics plan
**Maintenance (sustainment)**	Can the program be sustained over time? Across (a) settings and (b) patients	1. HCS Intent to continue or modify intervention following grant support 2. Patient medication adherence status 12 months after intervention is stopped 3. Can intervention be extended to other patient populations with different conditions and other settings?	1. Post-implementation qualitative interviews 2. Study database 3. D&I plan 4. Input from stakeholder panel

## Measures and analysis plan

### Reach analysis plan

We will use descriptive statistics to describe the following:

- Number of eligible patients and their baseline characteristics
- Percent of patients who did not opt out. We have also included a questionnaire for patients who opt out on reasons for declining. We will describe the patients who opt out and also return the questionnaire
- Percent of patients with a 7-day gap
- Representativeness of (a) study participants compared to overall patients within each clinic and respective health system and (b) of patients who opt out vs. those who do not on age, sex, number of conditions, ethnicity, and race

### Effectiveness analysis plan

The primary outcome is adherence to CV medications as measured by 12-month proportion of days covered (PDC), obtained using pharmacy records from each of the healthcare systems. Secondary outcomes include clinical events (e.g., event times for stroke, MI, mortality), utilization of care (e.g., hospitalizations or clinic visits for CV-related reasons), and costs of the interventions and of medical care, and will be captured from the EHR at each HCS. Subjects will be followed for 12 months after randomization to assess these primary and secondary outcomes. Subjects who have more than 1 year of follow-up (up to 3 years depending on when they are enrolled during years 2–3) will continue to be followed for secondary and longer-term maintenance outcomes. All analyses will be based on the intent to treat principle, using all patients who were randomized.

The assessment of primary outcome PDC will be based on the number of outpatient days a patient has a medication available, relative to the number of days during which a patient was prescribed the medication and should have depleted their supply, excluding inpatient days and days following death. The primary outcome will be average PDC, averaged across all medications the patient gapped on at baseline and is at risk of depleting on a day, and then averaged over all days when at risk of depleting at least one medication.

Descriptive analyses will be used to describe the cohort and to check for balance across study arms within strata (clinics and number of other medications prescribed). A simple ratio for primary outcome PDC will be calculated as the number of days at risk that the patient had medication divided by the number of days at risk, during the 1-year period following treatment initiation. These simple estimates of each patient’s PDC on each medication will be used for descriptive analyses. Missing patient covariate data will be imputed using multiple chained equations and multiple imputation [33].

Formal analyses will be based on daily data, using a day-level Bernoulli model with logistic link for the number of days covered by medication, which will be 365 but excluding days not at risk of depleting as described above. For a given medication, the model will include fixed effect terms for treatment arm, clinic, patient covariates, and a random subject effect for a subject’s tendency to have higher or lower PDC compared with other subjects. Patient covariates will include MyHealthConnect use (a medication reminder system used by some UCHealth patients), patient demographics (age, gender, race, ethnicity), number of clinic visits in the prior year, number of other CV medications the patient is prescribed at baseline, and indicators for major baseline CV conditions (AF, CAD, diabetes, hyperlipidemia, hypertension).

To estimate the means and treatment effects of PDC on a linear (PDC difference) scale, we will use methods of standardization (counterfactual calculations) [34] to estimate population average PDC for each treatment and each medication. With this approach, medication-specific models as above will be estimated using maximum likelihood and used to calculate the estimated probability a patient will have a given medication available on a given day, separately assuming they received each of the treatments, calculations which in some cases will be counterfactual. These estimated probabilities will be used to calculate two types of population average estimates: (a) medication-specific PDC for each treatment and (b) average PDC for each treatment across all medications gapped on at baseline. Each average is over all patients assuming they received each treatment, regardless of which treatment they actually received. Treatment differences will be estimated from the relevant quantities. Primary hypotheses involve pairwise comparisons between each of the four study arms and will be conducted using a multistage gatekeeper approach to control for multiple comparisons [35]. Inference will be carried out using bootstrap methods.

Secondary clinical outcomes will be analyzed using similar approaches but based on appropriate models, e.g., Cox survival models for time to clinical event or rehospitalization. Standardization methods again allow results to be expressed on interpretable scales such as risk difference [36]. Data will be analyzed using SAS (SAS Institute Inc., Cary, NC) and R.

We will use the methods described above and related methods to carry out additional analyses examining several types of moderation and mediation effects. We will use interactions to examine the heterogeneity of treatment effect (HTE) by drug class, healthcare system, and patient characteristics. We will also examine mechanisms or mediators of treatment effect by considering treatment effects on direct responses to reminders, including time from reminder to refill, number of 7-day gaps, and measures of patient engagement, e.g., number of patient text responses to reminders (intensive text and chatbot arms only).

#### Statistical power

The required sample size was estimated for the primary outcome 12-month PDC using preliminary data from the VA, based on the following assumptions: (a) two-sided level 0.05 tests, (b) power at least 80%, (c) difference between treatments in PDC of 10 percentage points, (d) Bonferroni adjustment for the 6 pairwise comparisons among the 4 study arms, (e) analysis stratified by healthcare system, and (f) within-system and within-treatment residual standard deviation of 12-month PDC equal to 0.22 (mean 0.732), obtained by analysis of 2,859 veterans during the period 01/01/2017–12/31/2017 who were prescribed relevant medications. With these assumptions, and comparing any two treatments using a simplified analysis based on a linear model with the above residual standard deviation of PDC, we estimate that we will need *N* = 119 subjects per treatment arm, total across the three healthcare systems, for a total of 476 subjects to be randomized across the three healthcare systems. To estimate available sample sizes, we obtained data from each of the three HCS on the number of patients at 4 VA, 5 UCHealth, and 8 DH clinics on estimated numbers of patients with CVD conditions and prescribed CVD medications. Assuming that 75% of patients have a gap, another 15% of patients opt out of the study following randomization, and 10% of patients do not have usable outcome data, we expect to have outcome data for about 7740 patients across the four study arms. Even with this conservative estimate, we expect to have ample subjects (nearly ten times as many as needed) to achieve the necessary power for the primary analysis of PDC. Additional subjects will provide power for secondary analyses, and for analyses of secondary outcomes (see Fig. 1).

### Adoption analysis plan

We will assess adoption in terms of the absolute number, proportion, and representativeness of the primary care clinics, physicians, and pharmacists that begin implementation of the intervention compared to all primary care clinics within each respective health system. We will compare the structural characteristics (e.g., staffing levels, number of providers, and number of patients) of clinics that participate in the intervention compared to all primary care clinics within the health system. We will also assess the absolute number, proportion, and representativeness of (a) providers and (b) pharmacists at a given clinic who have patients randomized to the intervention and compared to those who do not have any participating patients. These findings will help guide a dissemination campaign. Finally, we will conduct brief interviews with those staff who decline to identify reasons why.

### Implementation analysis plan

Implementation refers to the degree to which the intervention components and implementation strategies are implemented as intended, adaptations made, and costs of implementation (www.re-aim.org) [31]. Because the intervention is largely automated, we do not anticipate changes to intervention components. We still record any additions or modifications such as new or modified content to messages. It is more likely that some implementation strategies such as how providers are notified, exclusion criteria, how pharmacists interact with the nudge messages, and how this project fits into the work flow at each HCS will occur. Adaptations will be assessed using a modified FRAME adaptation model [37]. We will employ rapid, mixed methods assessment methods to assess specifics of issues such as the timing, type, purpose, and source of adaptations [38].

In our implementation analysis plan, for fidelity, we will address the issues outlined below. For most of these issues, we will use our study database and descriptive analyses (means, standard deviation, medians, and ranges) as well as analyses of variance to compare different subgroups and to answer the questions below.

- Among patients randomized to the intervention, how many text messages were delivered per patient, for which class of medication did the patient experience a gap, and did the patient have gaps on multiple medications during the course of the study
- Proportion of patients enrolled in text message versus IVR
- Among patients in arm #4 (optimized nudge plus AI chat bot), proportion where AI chat bot led to a patient response and the medication adherence barrier identified
- Barriers and facilitators to implementation of the intervention (see the “Key informant interviews” section below)
- Differences among the three HCS and different pharmacists
- Qualitative interviews focused on PRISM factors of (1) organizational and participants characteristics, (2) intervention characteristics from the organizational (healthcare system and providers) and participants’ perspectives (i.e., patients), (3) implementation and sustainability infrastructure (training and support), and (4) external environment

#### Economic analysis plan

This project includes two economic components. First, we will calculate the total cost of implementing each intervention to inform the resource use and investment required. Second, we will estimate the healthcare costs and cost offsets associated with the intervention arms to inform if there were reductions in healthcare utilization that resulted in overall cost savings.

To calculate the total *cost of implementing each intervention arm*, we will use a direct measure micro-costing approach. We will measure activities associated with the intervention and assign costs to them. Costs will be calculated by multiplying the number of units consumed by the unit cost for each cost component. Total costs will be stratified by upfront and implementation costs. Upfront costs will include those costs necessary to initiate the intervention, but occur before implementation. These will include the development of text and AI chat bot messages, translation of messages to Spanish, training of staff, etc. Implementation costs include those costs necessary to deliver the intervention. These may include the costs to send the text message to the randomized patients, the AI chat box services, etc.

Costs will be collected in a prospective fashion alongside the clinical trial and will include personnel and non-personnel costs. We have developed a log for involved personnel to record their time spent on intervention activities. The log captures resource use associated with the intervention and collects data on the activity that was done, who did the activity, the title of the person who did the activity, and how much time was spent on the activity. Non-personnel costs will be tracked through receipts and invoices paid. Personnel and non-personnel costs will be summed to generate the total cost of each intervention. All unit cost data will be adjusted to the same year US dollar through inflation and discounting. Costs will be stratified by cost type (upfront versus implementation), intervention arm, and HCS. The incremental intervention costs will be calculated by comparing each active intervention cost to the usual care cost. Pairwise comparisons between active interventions will also be calculated.

To estimate the *healthcare costs and cost offsets* associated with each intervention, EHR data from each HCS will be obtained on the healthcare utilization of patients in each study arm for a minimum of 12 months following implementation [34]. Using the same approach successfully employed in prior studies, we will extend the implications of this work by estimating healthcare costs from these utilization data. Once cost data are estimated from the utilization data using DRGs, RVUs, and AWP, they will be analyzed using the same models described for utilization and other study health outcomes with factors for the study arm and health system. Generalized linear models (GLM) will be used. The primary dependent variable will be healthcare cost, in total and separated into inpatient, outpatient, and pharmacy cost buckets. The primary independent variable will be the intervention arm the patient was randomized to. Results will be stratified by healthcare system.

The usual care arm will be the referent group and healthcare cost estimates for each active intervention arm will be compared to the usual care arm. If healthcare costs in an active intervention arm are significantly less than the healthcare costs in the usual care arm, as evidenced by a negative beta on the intervention arm coefficient, the active intervention will be associated with cost savings. Analysis of component 2 will be limited by the fact that utilization data will only be specific to healthcare utilization that occurred at each HCS. Therefore, we will not be able to examine the association of each intervention on total healthcare cost, but instead will be able to examine the association of each intervention on each HCS healthcare cost.

### Maintenance analysis plan

Maintenance analyses will use most of the same variables and methods as for effectiveness (for the individual level) and adoption (at the setting and staff levels) but at a later time period ranging from 1 to 23 months after July of 2021 when enrollment ends. The key questions to be answered are can the program be sustained over time across (a) settings, (b) patients, and (c) outcomes for patients. In addition, we will assess intent to continue or modify intervention following grant support, and if the intervention be extended to other settings patient populations with different contexts (see the “Dissemination plan” section below).

## Qualitative analysis

### Key informant interviews

We will conduct key informant interviews with up to 3 providers and 2 pharmacists (6–9 across the 3 HCS) from each setting whose patients received the intervention to get their feedback about the intervention and the intervention effects on their patient’s medication taking behavior. If there is less than 90% participation among either providers or pharmacists, we will also conduct phone interviews of those who decline to participate about reasons they declined. Many providers will have received one or more messages from the study team informing them that their patient did not refill their medications and we will interview the providers on their perceptions of that process. We will also conduct key informant interviews with HCS leaders (3–6 interviewees) in each setting who are responsible for institutional policies related to patient data management, informatics, and pharmacy. In these interactions, we will share findings from the research and gauge their reaction to the findings. With any indication of positive and efficient outcomes, we will ask participants to describe their likelihood to maintain the system within their setting, and to discuss any barriers to maintenance and specific actions needed to overcome these barriers or adapt the Nudge program in some way.

### Assessment of patient perspectives

**Fig. 5:**
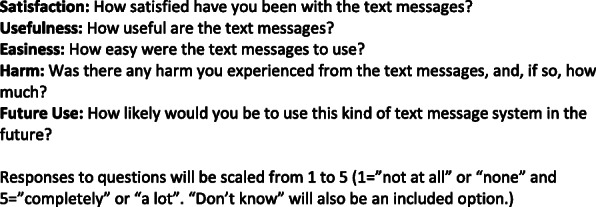
Example of text messaging survey

In year 4, after the intervention and follow-up period has ended, we will survey patients via text messaging using a previously developed text messaging survey (Fig. 5). In a random sample of 80 patients who respond to the survey, we will also conduct brief telephone interviews to get more in-depth feedback on the intervention. The sample will be stratified evenly across patients who received one of the three text messages. We have conducted similar interviews with patients following adherence interventions. These interviews will help inform further refinement of the interventions as we plan for broader dissemination (if demonstrated to be an efficient intervention) to more clinics and patients with other chronic conditions.

### Trial status

This is version 2 of the study protocol (as of 9/1/2020) registered with ClinicalTrials.org, and any further modifications to the protocol will be (a) shared with and approval obtained from the IRB and (b) communicated to the Data and Safety Monitoring Board. Patient recruitment began Nov. 11, 2019, and is estimated to be completed approximately July 30, 2022.

## Dissemination plan

There are two key aspects to our dissemination plan, both informed by the recent literature and our team’s work on designing for dissemination [28–30, 39–42]. (1) We conduct all activities including planning and stakeholder engagement activities with a focus on both (a) continued use after our intervention and evaluation activities have concluded and (b) eventual use in diverse settings, “designing for dissemination” from the outset, and updating/refining our plans throughout the project. (2) We will conduct different dissemination product development activities and then implement communication vehicles for each of our target audiences.

We have two primary target audiences: (1) HCS that could potentially adopt our Nudge program intervention components and implementation strategies that prove most efficient and (2) medication adherence and illness self-management research or quality improvement teams that could use, replicate, and extend findings using our protocols and resources.

For HCS, we will develop “implementation and adaptation guides” (sometimes referred to as playbooks) to help settings potentially interested in adopting our Nudge intervention (or its key components and core functions) in their system. Based on our ongoing experience in our Triple Aim QUERI program [42] developing such guides for different health system change projects, we will develop interactive “living documents” [43]. In years 3–4, we will then pilot implementation of these resources to allow health systems to decide if this program could fit their HCS at the present time, and if so how they can (a) ensure that key program elements and functions are delivered consistently and (b) that necessary and appropriate adaptations are made to make the program viable in their settings. In year 4 and follow-up grants, we will then fully implement these dissemination/adaptation guides.

Details of this process of developing interactive “living adaptation guides” are discussed in the section below. We will partner with our organizational stakeholders to identify the best dissemination venues and methods to reach this target audience. We expect these venues to include already existing meetings such as national community health centers, managed care, VA regional and national meetings, and national professional organization conferences that our target audience already frequents. These guides will reflect adaptations made over the course of the project as well as interviews with program implementers toward the conclusion of their intervention period. Adaptations will be identified via both interviews and tracking forms during years 2 and 3 [32, 39, 44] but will likely include technologic adaptations owing to the differing nature of site pharmacy refill data, new or different EHR systems or features, considerations for clinical follow-up processes, altered language in text messages to reflect local culture, and varying procedures to inform patients that they are involved in the messaging program.

### Development of HCS implementation and adaptation guide

We will develop web-based implementation and adaptation guides that will compile the evidence-based findings from our research to support dissemination and implementation of the Nudge program in new settings. We will identify the core functions of the intervention as well as adaptable components [32, 39, 44, 45] during years 2–3. First, we will create a scalability guide, focused on scaling up the intervention from a modest number of clinics within each of the three participating healthcare systems to system-wide implementation. This toolkit will include detailed steps to (1) identify nonadherent patients, (2) create linkages to text messages and/or AI chat bot through Twillio and/or Textit, and (3) steps to download implementation data that will allow for ongoing audit and feedback. This guide can also be used to scale out for other HCS wishing to (a) decide if this program is right for them (with or without adaptation), (b) implement a Nudge program, or (c) apply our principles and procedures for other healthcare issues.

The adaptation portion of the guide will offer guidance on how to use our theoretical frameworks for message design and optimization to create and pilot test messages relevant for other conditions and/or other medications. It will include examples of text messages, generic or behavioral nudges, examples of chat bot conversations with patients, and examples of EHR notes to clinicians informing them that their patient has not refilled their medication. Similar to the guides we have developed for our QUERI projects, the guide will also include materials and templates reflecting the content of each intervention component, action-oriented recommendations, Frequently Asked Questions, guidance for future adaptations, resources required, and tools that have been found to facilitate successful implementation of the intervention. The final adaptation guides will be employed during year 4 for hypertension medication adherence and then in a follow-up grant, tested with other conditions.

### Dissemination to research teams

Our second dissemination goal will be to enhance the science of dissemination research and to disseminate to *research teams* by transparently reporting our protocol in clincicaltrials.gov and the journal *Implementation Science*. In later years, we will publish our implementation results and lessons learned using the PRISM and RE-AIM frameworks in journal articles and present our findings at targeted national meetings including the annual NIH Dissemination and Implementation in Health meeting, the Academy Health annual research meeting, the Society of Behavioral Medicine meeting; relevant professional society meetings; and national and regional VA and HCS research meetings.

In addition, we will offer seminars, demonstrations, and workshops that train other research teams in how to successfully implement and evaluate a Nudge program in their setting. Finally, we will share these materials with the other sites in the NIH Collaboratory, working with them and the coordinating center to make all scientific and pragmatic information available in ways that can be tailored for use in other sites.

## Discussion

Two crosscutting approaches will be employed across our project that provide both great potential and likely operational challenges. These involve designing for dissemination (D4D) principles [26, 41] and related approaches to enhance sustainment. Our D4D efforts will consist of (1) user-centered rapid and iterative design methods [46, 47] which are employed in Dr. Bull’s laboratory and (2) significant and ongoing multi-level stakeholder engagement.

### User-centered design thinking and rapid development procedures

We employed persuasive communication strategies with demonstrated efficacy for engaging participants in the social media realm. Critical to our iterative, user-centered design procedures were consideration of message design to maximize access, which required attention to user literacy and numeracy. All messages were kept at or below a 5th grade reading level [48]. To this end, we spent 3 months in developing our message libraries for each intervention arm.

To ensure adequate involvement of potential users, we purposively sampled 20 participants from each of the three participating HCS—VA, DH and UCHealth—with a balance of older/younger patients, men/women, those with one versus multiple chronic CV conditions, and native Spanish/English speakers. We generated and tested both English and Spanish text messages and framed chat bot communications through multiple *N* of 1 (i.e., within subject) assessments that conformed to evidence-based strategies for persuasive message design [49–52]. This approach offered a way to quickly respond and iterate new versions of messages until consensus across participants, including for perceived efficacy and general acceptability, was reached. In later project years, we will use similar methods to develop and pilot test additional implementation strategies as needed to maximize adherence.

#### Stakeholder engagement

Significant and ongoing consumer involvement facilitates methods for including the perspective of potential adopting settings and implementation staff, as well as patients receiving the targeted services. We developed a stakeholder group with representation from each of our three HCS and included primary care clinicians, pharmacists/pharmacy staff, clinical operations directors, local IT staff, and patients being treated for at least one of the five CV diagnoses of interest. There are three dimensions along which we have and will continue to maximize stakeholder engagement. First is the level of engagement as our stakeholders were involved in the proposal development and will be in significant ongoing ways integral to the project. Stakeholders are treated as members of the research team and engaged in an ongoing, iterative manner, answering conceptual, ethical, and operational questions through their participation. Importantly, stakeholders will continue to be involved throughout the life of the Nudge project and will contribute to the initial design, rapid prototyping and subsequent iterations of the intervention, review of emerging results, and development of communication and dissemination materials. Finally, we engage multiple types of stakeholders—not just patients and high-ranking organizational officials. We include (a) patients and their families or significant others, (b) “implementers” or staff such as care managers and pharmacists that directly interact with patients and support the Nudge interventions, and (c) decision-makers. Specific stakeholders involved will vary over time and include different types of representatives—early on, leaders responsible for deciding upon and gaining support for the program; during the middle stages, IT staff and supervisors responsible for deciding upon IT priorities and integrating Nudge with other HCS electronic technologies; and toward the later stages, those responsible for sustained funding, training, implementing Nudge, and supervising relevant staff.

### Strengths, limitations, and future directions

#### Limitations

This project has both strengths and limitations. Limitations include that the three HCS are all integrated care systems, with some degree of centralized pharmacy services. Our results may not apply to less integrated HCS. Two strategies that we used to make the project more pragmatic: having minimal exclusion criteria at multiple levels (physicians, pharmacists, and patients) and having strong, multi-level stakeholder engagement may increase the variability of interventions, implementation strategies, and results, making it more challenging to detect significant effects. Stakeholder engagement could, like our decision to allow adaptations to each HCS, result in the study being less efficient, enhance variability, or reduce “fidelity” to the intervention. This said, we think these were correct decisions since our goal was to design and evaluate a pragmatic, feasible, cost-efficient intervention likely to appeal to and involve different HCS and be more likely to be maintained after the study period.

#### Strengths and future directions

The strengths of the project, which to some extent mirror the limitations above, are working with different HCS and the heterogeneity of patient populations; stakeholder engagement to tailor strategies to local settings; allowing, assessing, and transparently reporting adaptations; and the generally pragmatic design. We are employing multi-level conceptual models (Expanded RE-AIM/ PRISM), transparent reporting criteria, and addressing key implementation science issues such as adaptations and sustainability. In addition, unique aspects of the project include the large-scale use of interactive nudge technologies, the focus on pharmacists rather than relying solely on physicians, the economic analyses, and explicit designing for dissemination and sustainability. The results of this investigation should provide a strong basis for follow-up studies including broader dissemination to different HCS, comparative effectiveness research on variants of the most efficient interventions and implementation strategies identified, applications to different chronic conditions, and evaluation of long-term sustainment.

## Supplementary Information

**Additional file 1.** Nudge Opt out letter

## Data Availability

The datasets used in the current paper (e.g., estimates for the CONSORT figure and the PRECIS-2 individual ratings) are available from the senior author on reasonable request. Study results are not yet available as this is a protocol paper.
