# Supplementary material for: The NUDGE trial pragmatic trial to enhance cardiovascular medication adherence: study protocol for a randomized controlled trial
Source: Trials. 2021 Aug 11;22:528. doi: 10.1186/s13063-021-05453-9 (PMC8356469; doi:10.1186/s13063-021-05453-9)
Supplement: Supplementary file 1 — Additional file 1. Nudge Opt out letter [file 13063_2021_5453_MOESM1_ESM.docx]

12/05/20

Updated Personal cover letter

We have now made the changes instructed in the latest communication. Specifically, we have:

1. Added a Trial Status section immediately after Figure 5 in the text
2. Moved all the declarations before the references.

Original cover letter:

We think this paper, Pragmatic Trial to Enhance Medication Adherence: Protocol for the NUDGE Trial, is appropriate for *Trials* because it describes a rigorous large-scale evaluation of an efficient, pragmatic intervention to enhance medication adherence for a variety of cardiovascular conditions. The protocol includes individual level randomization, assessment of implementation outcomes using the RE-AIM framework, well specified multi-level outcomes, and an economic evaluation across three different large health care systems.

We believe that our paper meets all the requirements for a protocol publication with the exception of one potential issue. Specifically, our IRB approval was approximately 14 months prior to this submission. The predominant reason for this delay was due to ramifications of the COVID-19 pandemic.

All authors have approved submissions of the manuscript, there are no competing or conflicts of interest, and we confirm that the manuscript has not been published or submitted elsewhere.
